# The impact of ethnic background on ICU care and outcome in sepsis and septic shock – A retrospective multicenter analysis on 17,949 patients

**DOI:** 10.1186/s12879-023-08170-7

**Published:** 2023-03-31

**Authors:** Andreas Koköfer, Behrooz Mamandipoor, Maria Flamm, Richard Rezar, Sarah Wernly, Christian Datz, Christian Jung, Venet Osmani, Bernhard Wernly, Raphael Romano Bruno

**Affiliations:** 1 Department of Anaesthesiology, Perioperative Medicine and Intensive Care Medicine, Paracelsus Medical University of Salzburg, Salzburg, Austria; 2 Institute of General Practice, Family Medicine and Preventive Medicine, Paracelsus Medical University of Salzburg, Salzburg, Austria; 3 Fondazione Bruno Kessler Research Institute, Trento, Italy; 4 Department of Cardiology, Paracelsus Medical University of Salzburg, Salzburg, Austria; 5 Department of Internal Medicine, General Hospital Oberndorf, Teaching Hospital, Paracelsus Medical University of Salzburg, Oberndorf, Austria; 6 Division of Cardiology, Pulmonology and Vascular Medicine, Medical Faculty, University Hospital Düsseldorf, Heinrich-Heine-University Düsseldorf, Düsseldorf, Germany

**Keywords:** Sepsis, Intensive care, Critically ill, Elderly, Very elderly, Old, Very old, Octogenarian, Geriatric

## Abstract

**Background:**

Previous studies have been inconclusive about racial disparities in sepsis. This study evaluated the impact of ethnic background on management and outcome in sepsis and septic shock.

**Methods:**

This analysis included 17,146 patients suffering from sepsis and septic shock from the multicenter eICU Collaborative Research Database. Generalized estimated equation (GEE) population-averaged models were used to fit three sequential regression models for the binary primary outcome of hospital mortality.

**Results:**

Non-Hispanic whites were the predominant group (*n* = 14,124), followed by African Americans (*n* = 1,852), Hispanics (*n* = 717), Asian Americans (*n* = 280), Native Americans (*n* = 146) and others (*n* = 830). Overall, the intensive care treatment and hospital mortality were similar between all ethnic groups*.* This finding was concordant in patients with septic shock and persisted after adjusting for patient-level variables (age, sex, mechanical ventilation, vasopressor use and comorbidities) and hospital variables (teaching hospital status, number of beds in the hospital)*.*

**Conclusion:**

We could not detect ethnic disparities in the management and outcomes of critically ill septic patients and patients suffering from septic shock. Disparate outcomes among critically ill septic patients of different ethnicities are a public health, rather than a critical care challenge.

## Background

**Fig. 1:**
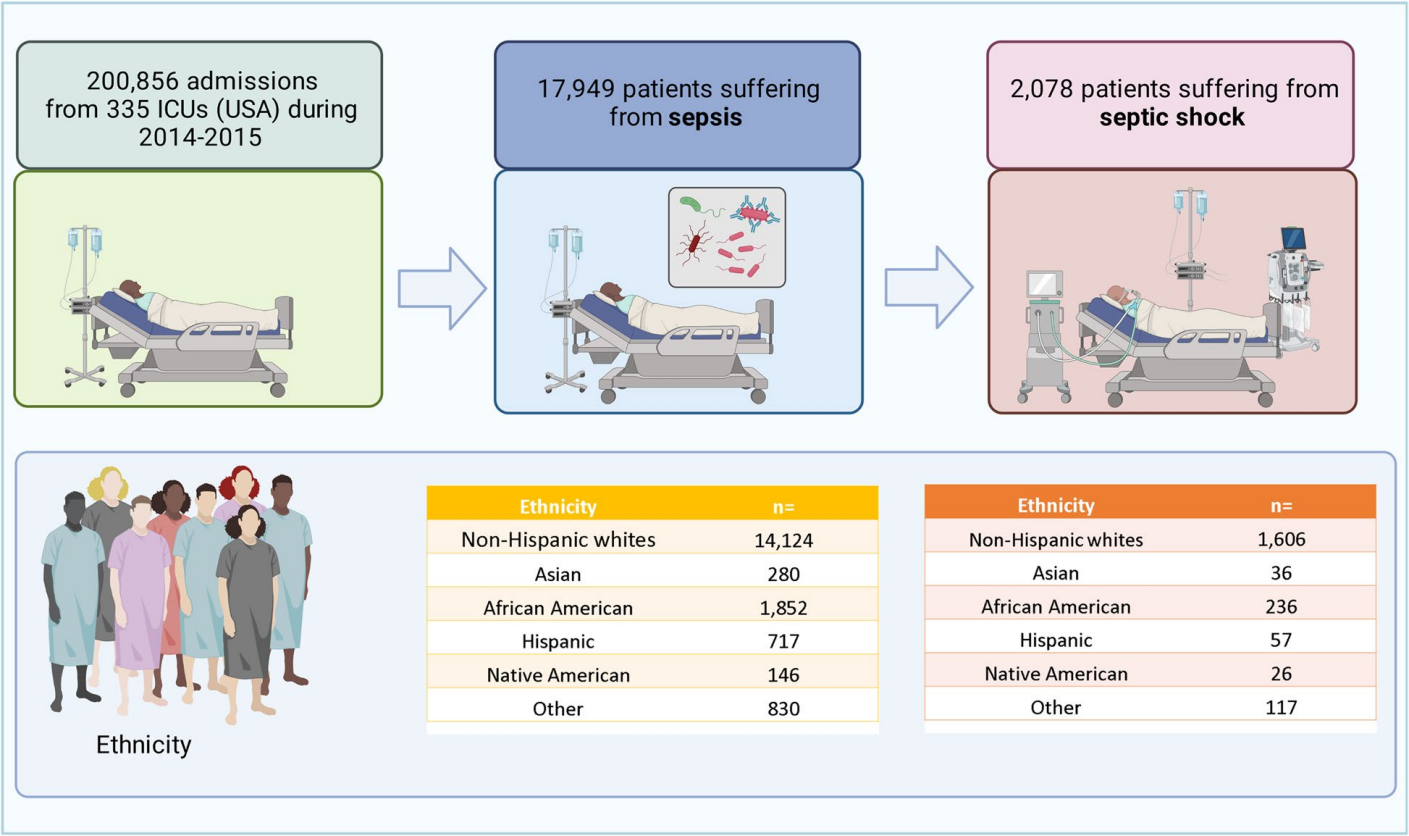
Consort Diagram

**Fig. 2:**
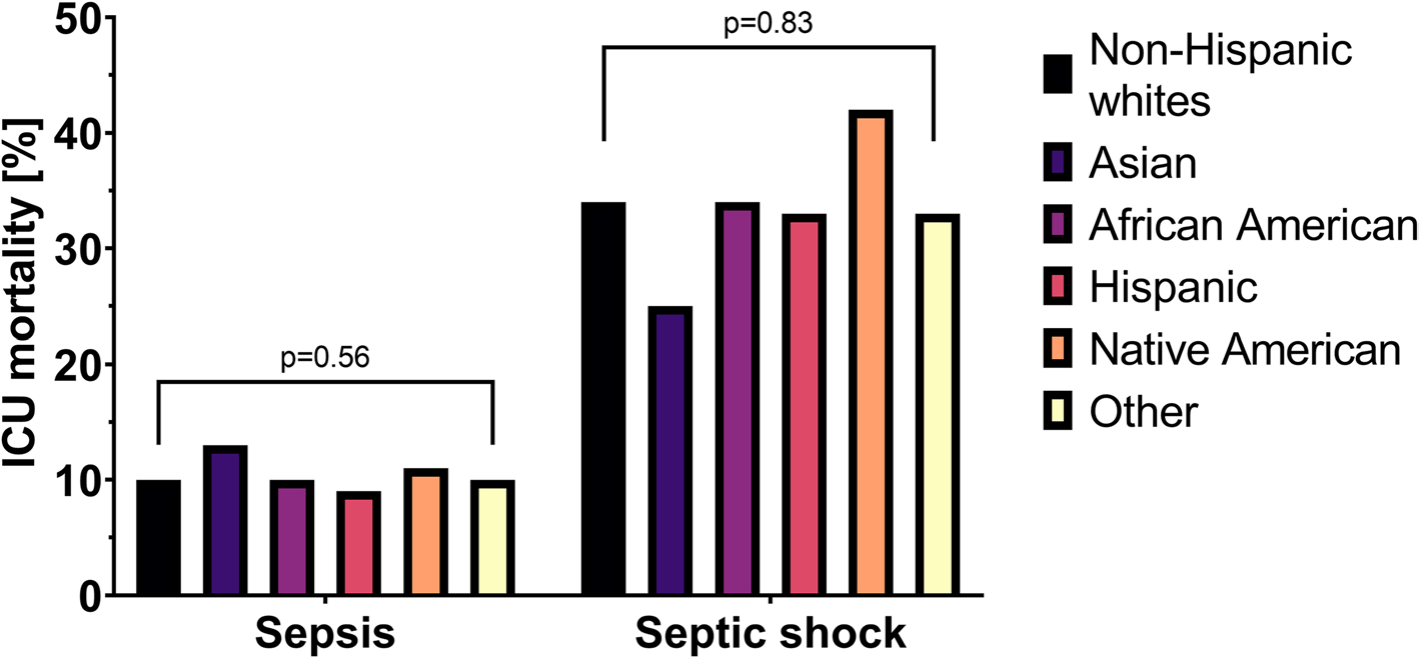
Primary outcome intensive care unit (ICU) mortality [%]

A pronounced phenomenon of ethnic differences in medical care and outcomes has been described in many areas of medicine [1]. Racial disparities in critical care, particularly affecting African American patients, have been documented in multiple studies [2–5]. These multidimensional disparities affect various racial groups [6–8]. The persistence of such discrepancies in critical care is surprising, given that critical care is an area of medicine with clearly defined indications and standardized interventions. However, studies have demonstrated ethnic disparities in critical care, not only in treatment and underlying causes [9–13], including differences in the incidence of sepsis in age- and sex-standardized African American populations compared to Caucasian Americans [14, 15]. These differences are alarming as sepsis still has one of the highest mortality rates in all conditions requiring critical care, upwards of 45% to 60% in patients with septic shock [15]. While the presence of ethnic disparities in the management of sepsis was commonly established in past literature, more recent studies have produced seemingly contradictory results [16]. Vazquez Guillamet et al. demonstrated that socioeconomic rather than ethnic background is critical in the management and outcomes of critically ill patients with sepsis [17]. It is comprehensible that hospitals serving predominantly minority population may have poorer outcomes., due to their location, funding, and insurance status of patients, However, it is still unclear to what extent patients of minority lineage have worse outcomes within the same institutions. Therefore, this study aimed to investigate the impact of different ethnic backgrounds on intensive care treatment and hospital mortality in sepsis and septic shock in eICU, one of the most extensive datasets of critically ill patients (Figs. 1 and 2).

## Methods

This analysis included 17,949 patients with diagnosed sepsis, according to the Acute Physiology and Chronic Health Evaluation (APACHE) IV [18]. Septic shock was defined as a diagnosis of sepsis through APACHE IV, vasopressor requirement, and serum lactate level greater than 2 mmol/L. Data was obtained from the multicenter eICU Collaborative Research Database, which includes over 200,859 admissions of 335 intensive care units (ICUs) from 208 hospitals across the USA in 2014 and 2015. The dataset has been described previously [19]. The database is released under the Health Insurance Portability and Accountability Act (HIPAA) safe harbor provision. As described previously, we extracted the baseline characteristics and organ support on day one [20–22].

### Statistical analyses

We expressed continuous data points as median ± interquartile range and assessed differences between independent groups using the Kruskal–Wallis equality-of-populations rank test. Categorical data was stated in numbers (percentage) and we calculated univariate differences between groups using the Chi-square test. The primary exposure was the ethnic group. The primary outcome was ICU mortality. Secondary outcomes were the length of stay in the ICU, the frequencies of mechanical ventilation and vasopressor use. We used a generalized estimated equation (GEE) and population-averaged models to fit three sequential regression models for the binary primary outcome to evaluate the impact of the ethnic group on hospital mortality. First, a baseline model with the ethnic group as a fixed effect and hospital mortality as a random effect (model-1) was fitted. The baseline model was expanded to included patient level characteristics as independent variables (model-2). (BMI, SOFA score, gender, Elixhauser comorbidity score [23]). Model-2 was further augmented to include … (model-3). Third, to model-2, hospital variables (teaching hospital status, number of beds in hospital) were added to the model (model-3). We chose the independent variables based on our clinical experience and previous studies. We obtained adjusted odds ratios (aOR) with 95% confidence intervals (95%CI) for all three models. All tests were two-sided, and a *p*-value of < 0.05 was considered statistically significant. We used Stata/IC 16.1 (StataCorp. 2019. Stata Statistical Software: Release 16. College Station, TX: StataCorp LLC) for all the statistical analyses.

## Results

Patients were categorized as Non-Hispanic whites (*n* = 14,124), African American (*n* = 1,852), Hispanic (*n* = 717), Asian American (*n* = 280), Native American (*n* = 146) and Others (*n* = 830). Non-Hispanic white patients were the most frequent and defined as a reference category compared to all other ethnicities (*n* = 3,825). The six ethnic groups differed regarding their baseline characteristics (Table 1). Native Americans were significantly younger (mean 55 years, SD 46–65 years, *p* < 0,001) than the other groups. Consecutively the number of very old patients (> 80 years) was lowest in the Native American group being only 9% (*n* = 13), compared to the Hispanic group with the highest percentage of octogenarians (28% (*n* = 203), *p* < 0.01). There were no differences in gender and BMI. Although the APACHE score was without statistical differences between the groups, the SOFA score and the creatinine levels on day 1 (creatinine being a major contributor to the SOFA score) were statistically significantly higher in the African American subgroup than in all other ethnicities (mean 1.7 mg/dL, SD 1.0–3.5 mg/dL vs. mean 1,3 mg/dL, SD 0.9–2.3 mg/dL, *p* < 0.001). In contrast, lactate on admission was significantly higher in Asian patients (2.3 mmol/L, SD 1.5–3.9 mmol/L) and lowest in Native Americans (1.9 mmol/L, SD 1.1–3.4 mmol/L, *p* = 0.01). Correspondingly, the percentage of patients with baseline lactate greater than 2 mmol/L was higher in Asian patients than in all other groups. Notably, African Americans evidenced significantly lower hemoglobin on admission (9.7 g/dL, SD 8.3–11.1 g/dL, *p* < 0.001), while platelets were lowest in Native Americans (168.5 times 1000, SD 84.0–227.0 times 1000, *p* < 0.001).

**Table 1:** Baseline characteristics of critically ill patients suffering from sepsis

	**Non-Hispanic whites**	**Asian**	**African American**	**Hispanic**	**Native American**	**Other**	*p*-value
	*N* = *14,124*	*N* = *280*	*N* = *1,852*	*N* = *717*	*N* = *146*	*N* = *830*	
Age (years)	68 (57–79)	66 (55–79)	62 (51–73)	68 (51–81)	55 (46–65)	65 (51–77)	< 0.001
Male	50% (7,097)	48% (135)	54% (995)	49% (351)	43% (63)	55% (454)	0.045
BMI	27 (23–33)	25 (21–28)	27 (22–33)	26 (22–30)	28 (24–37)	27 (23–31)	< 0.001
SOFA	5 (3–8)	6 (3–8)	6 (4–9)	5 (3–8)	6 (4–10)	6 (3–9)	< 0.001
APACHE	65 (50–82)	63 (45–82)	66 (49–85)	65 (49–83)	69 (52–90)	66 (47–86)	0.16
Lactate on admission [mmol/L]	1.9 (1.2–3.2)	2.3 (1.5–3.9)	2.0 (1.2–3.7)	2.0 (1.3–3.7)	1.9 (1.1–3.4)	2.0 (1.3–3.5)	0.010
Lactate on admission > 2 mmol/L	42% (3,732)	51% (90)	45% (500)	43% (180)	43% (46)	46% (250)	0.039
Serum creatinine on admission [mg/dL]	1.3 (0.9–2.3)	1.2 (0.8–2.3)	1.7 (1.0–3.5)	1.2 (0.8–2.0)	1.3 (0.7–2.9)	1.3 (0.8–2.3)	< 0.001
Hemoglobin (g/dL)	10.3 (8.9–11.8)	10.3 (8.7–11.8)	9.7 (8.3–11.1)	10.2 (8.8–11.6)	10.1 (8.2–11.6)	10.3 (8.9–11.7)	< 0.001
Platelets × 1000	183.0 (127.0–254.0)	170.5 (114.0–235.0)	186.0 (124.0–258.0)	178.0 (118.0–250.0)	168.5 (84.0–227.0)	176.0 (119.0–245.0)	< 0.001
WBC × 1000	12.9 (8.5–18.6)	11.5 (7.6–17.6)	12.5 (8.2–18.9)	12.2 (8.0–18.2)	14.6 (8.7–21.1)	12.6 (8.2–18.5)	0.056
Primary focus							< 0.001
GI	12% (1,759)	14% (38)	8% (157)	13% (93)	14% (20)	14% (116)	
Cutaneous/soft tissue	8% (1,184)	9% (25)	9% (173)	6% (43)	14% (21)	6% (50)	
Gynecologic	0% (33)	0% (1)	0% (8)	1% (8)	1% (1)	1% (5)	
Other	6% (787)	7% (20)	10% (189)	12% (86)	3% (5)	8% (64)	
Pulmonary	39% (5,505)	34% (95)	33% (608)	28% (200)	34% (50)	36% (301)	
Renal/UTI (including bladder)	23% (3,315)	20% (56)	24% (436)	28% (200)	26% (38)	25% (208)	
Unknown	11% (1,541)	16% (45)	15% (281)	12% (87)	8% (11)	10% (86)	

*BMI* body mass index, *SOFA* sequential organ failure assessment, *WBO* white blood count, *GI* gastrointestinal, *UTI* urinary tract infection

Pneumonia was, in absolute numbers, the most common septic focus in all patients (*n* = 6,759), followed by renal infections/UTI (urinary tract infection) (*n* = 4,253) and GI infections (*n* = 2,183). However, the source of infections differed significantly: For example, Hispanic patients had more UTI, while Native Americans were more affected by cutaneous and soft tissue infections. Statistically significant differences were not observed in mechanical ventilation, vasopressors, and renal replacement therapy frequency during intensive care treatment. (Table 2). African Americans had a significantly longer stay in the ICU (55 h, SD 29-112 h), Hispanic and Native American patients shortest (49 h, SD 26-89 h, and 49 h, SD 27-93 h, *p* = 0.031). Neither ICU nor hospital mortality differed between the ethnic groups. Although statistically not significant, the Asian American subgroup tended to have the highest hospital (19%, *p* = 0.68) and ICU mortality (13%, *p* = 0.56). These findings persisted after multivariable adjustment in the GEE sequential regression analyses for hospital mortality (Table 3).

**Table 2:** ICU interventions, therapies and outcomes of septic patients

	**Non-Hispanic whites**	**Asian**	**African American**	**Hispanic**	**Native American**	**Other**	*p*-value
	*N* = *14,124*	*N* = *280*	*N* = *1,852*	*N* = *717*	*N* = *146*	*N* = *830*	
Mechanical ventilation	22% (3,097)	19% (54)	22% (408)	19% (137)	27% (40)	24% (203)	0.072
Vasopressor use	32% (4,488)	30% (84)	32% (584)	33% (234)	39% (57)	33% (273)	0.46
RRT	3% (362)	1% (3)	3% (55)	2% (12)	4% (5)	4% (30)	0.062
LOS (h)	53 (29–99)	50 (30–104)	55 (29–112)	49 (26–89)	49 (27–93)	53 (25–107)	0.031
LOS > 7 days	12% (1,715)	13% (36)	14% (262)	12% (84)	11% (16)	13% (107)	0.23
Hospital mortality	16% (2,268)	19% (52)	16% (304)	16% (117)	16% (24)	14% (120)	0.68
ICU mortality	10% (1,449)	13% (36)	10% (186)	9% (63)	11% (16)	10% (87)	0.56

*ICU* intensive care unit, *RRT* renal replacement therapy, *LOS* length of stay

**Table 3:** Generalized estimated equation (GEE), population-averaged sequential regression analyses for hospital mortality *for patients suffering from sepsis* (aOR *(95%CI, p-value))*

*Non-Hispanic whites* = *Reference (aOR / OR* = *1.0)*			
	**Model-1**	**Model-2**	**Model-3**
**Asian**	1.22 (0.94–1.58, *p* = 0.126)	1.30 (0.95–1.80, *p* = 0.105)	1.20 (0.86–1.67, *p* = 0.296)
**African American**	1.01 (0.84–1.22, *p* = 0.913)	0.91 (0.75–1.10, *p* = 0.341)	0.94 (0.78–1.15, *p* = 0.571)
**Hispanic**	0.96 (0.79–1.17, *p* = 0.718)	0.94 (0.73–1.22, *p* = 0.656)	0.89 (0.69–1.16, *p* = 0.395)
**Native American**	0.92 (0.61–1.41, *p* = 0.712)	0.92 (0.59–1.44, *p* = 0.726)	0.91 (0.57–1.45, *p* = 0.686)
**Other**	0.93 (0.74–1.16, *p* = 0.501)	0.86 (0.68–1.09, *p* = 0.208)	0.86 (0.67–1.10, *p* = 0.231)

*Model—1:* Ethnicity as fixed and individual ICU as random effect

*Model—2:* Model -1 plus SOFA, gender, age, Elixhauser comorbidities

*Model—3:* Model -2 plus teaching hospital status and number of beds in hospital

The baseline characteristics among the subgroup of patients suffering from septic shock are displayed in Table 4. Again, Native Americans were significantly younger (53 years, SD 44–61 years, *p* < 0.001) than the other ethnic groups, but they demonstrated the highest SOFA scores (14, SD 8–16, *p* < 0.001). There were no differences in the intensive care treatment (Table 5) in the subgroup of patients in septic shock: Asian Americans had the most prolonged stay at the ICU (110 h, SD 72-205 h, *p* = 0.008), although their mortality was lowest (25% for the ICU mortality and 28% for the hospital mortality, *p* = 0.83 and *p* = 0.31, respectively). The baseline characteristics and the overall ICU and hospital mortality rates of patients in septic shock are displayed in Tables 4 and 5. The multilevel GEE sequential regression analysis for hospital mortality found no differences between the ethnic groups regarding the primary endpoint of hospital mortality (Table 6). Additionally, there was no statistically significant difference in mortality between ethnic groups in patients with septic shock.

**Table 4:** Baseline characteristics of patients suffering from septic shock

	**Non-Hispanic whites**	**Asian**	**African American**	**Hispanic**	**Native American**	**Other**	*p*-value
	*N* = *1,606*	*N* = *36*	*N* = *236*	*N* = *57*	*N* = *26*	*N* = *117*	
Age (years)	68 (58–78)	62 (47–73)	65 (56–77)	68 (54–83)	53 (44–61)	65 (53–76)	< 0.001
Male	52% (842)	47% (17)	53% (126)	44% (25)	46% (12)	54% (63)	0.76
BMI	27 (23–33)	24 (21–28)	27 (22–32)	26 (22–30)	30 (23–40)	27 (23–32)	0.004
SOFA	10 (7–12)	8 (7–12)	11 (8–13)	9 (7–12)	14 (8–16)	11 (8–13)	< 0.001
APACHE	88 (70–110)	85 (62–107)	97 (74–120)	88 (72–107)	100 (85–129)	93 (73–115)	0.002
Lactate on admission [mmol/L]	4.2 (2.9–7.2)	4.2 (2.5–7.5)	4.7 (3.1–8.7)	5.3 (3.4–7.8)	4.8 (3.4–8.1)	4.7 (3.2–7.3)	0.036
Lactate on admission > 2 mmol/L	100% (1,606)	100% (36)	100% (236)	100% (57)	100% (26)	100% (117)	
Serum creatinine on admission [mg/dL]	2.0 (1.3–3.0)	1.5 (1.0–3.0)	2.5 (1.6–4.1)	1.8 (1.2–2.8)	2.3 (1.1–3.0)	2.2 (1.2–3.2)	< 0.001
Hemoglobin (g/dL)	10.4 (8.9–12.0)	11.0 (8.4–12.0)	9.6 (8.4–11.1)	9.8 (8.5–11.4)	9.8 (8.1–12.1)	10.1 (8.9–11.5)	< 0.001
Platelets × 1000	154.5 (95.0–234.0)	150.5 (110.0–190.0)	160.5 (99.5–234.5)	121.0 (65.0–213.0)	100.5 (50.0–180.0)	129.5 (71.0–206.0)	0.004
WBC × 1000	15.6 (9.4–22.6)	16.1 (8.8–24.5)	15.4 (8.4–23.0)	15.0 (7.0–24.2)	12.6 (4.2–21.3)	13.8 (6.0–23.0)	0.56
Primary focus							< 0.001
GI	19% (305)	19% (7)	9% (22)	19% (11)	19% (5)	20% (23)	
Cutaneous/soft tissue	6% (103)	11% (4)	8% (19)	0% (0)	4% (1)	3% (4)	
Gynecologic	0% (4)	0% (0)	0% (1)	4% (2)	0% (0)	1% (1)	
Other	6% (102)	14% (5)	15% (36)	9% (5)	0% (0)	5% (6)	
Pulmonary	34% (551)	19% (7)	29% (69)	33% (19)	38% (10)	40% (47)	
Renal/UTI (including bladder)	21% (331)	19% (7)	20% (47)	19% (11)	27% (7)	16% (19)	
Unknown	13% (210)	17% (6)	18% (42)	16% (9)	12% (3)	15% (17)	

*BMI* body mass index, *SOFA* sequential organ failure assessment, *WBO* white blood count, *GI* gastrointestinal, *UTI* urinary tract infection

**Table 5:** ICU interventions, therapies and outcomes of patients in septic shock

	**Non-Hispanic whites**	**Asian**	**African American**	**Hispanic**	**Native American**	**Other**	*p*-value
	*N* = *1,606*	*N* = *36*	*N* = *236*	*N* = *57*	*N* = *26*	*N* = *117*	
Mechanical ventilation	52% (834)	50% (18)	54% (128)	47% (27)	58% (15)	56% (65)	0.87
Vasopressor use	100% (1,606)	100% (36)	100% (236)	100% (57)	100% (26)	100% (117)	n/a
RRT	6% (84)	0% (0)	5% (10)	4% (2)	4% (1)	7% (7)	0.66
LOS (h)	71 (36–150)	110 (72–205)	99 (40–196)	84 (40–187)	94 (31–184)	76 (40–201)	0.008
LOS > 7 days	22% (352)	28% (10)	29% (69)	30% (17)	31% (8)	27% (32)	0.077
Hospital mortality	43% (683)	28% (10)	46% (108)	40% (23)	54% (14)	40% (47)	0.31
ICU mortality	34% (544)	25% (9)	34% (80)	33% (19)	42% (11)	33% (39)	0.83

*ICU* intensive care unit, *RRT* renal replacement therapy, *LOS* length of stay

**Table 6:** Generalized estimated equation (GEE), population-averaged sequential regression analyses for hospital mortality *for patients suffering from septic shock (aOR (95%CI, p-value))*

*Non-Hispanic whites* = *Reference (aOR / OR* = *1.0)*			
	**Model-1**	**Model-2**	**Model-3**
**Asian**	0.53 (0.25–1.14, *p* = 0.104)	0.60 (0.28–1.33, *p* = 0.208)	0.57 (0.24–1.36, *p* = 0.206)
**African American**	1.13 (0.82–1.56, *p* = 0.451)	0.99 (0.70–1.40, *p* = 0.946)	1.01 (0.72–1.43, *p* = 0.937)
**Hispanic**	0.97 (0.53–1.78, *p* = 0.925)	0.92 (0.45–1.89, *p* = 0.83)	0.80 (0.37–1.70, *p* = 0.554)
**Native American**	1.68 (1.05–2.67, *p* = 0.029)	1.50 (0.84–2.67, *p* = 0.170)	1.51 (0.84–2.70, *p* = 0.168)
**Other**	0.99 (0.66–1.47, *p* = 0.942)	0.92 (0.60–1.42, *p* = 0.719)	0.95 (0.61–1.49, *p* = 0.834)

*Model—1:* Ethnicity as fixed and individual ICU as random effect

*Model—2:* Model -1 plus SOFA, gender, age, Elixhauser comorbidities

*Model—3:* Model -2 plus teaching hospital status and number of beds in hospital

## Discussion

This multicenter study, which included 17,949 patients, found no impact of ethnic background on the hospital mortality of septic patients in the ICU. This finding was consistent in all evaluated sub-groups and after multivariable adjustment for patient-level characteristics and hospital variables. While preliminary studies have shown mortality differences among different ethnicities, our findings may appear contradictory [11, 24–28]. However, our finding is well supported by a more recent analysis of a smaller cohort by Vazquez Guillamet et al. [17]. Several considerations exist for conducting any ethnicity-specific investigation in critically ill patients: Firstly, structural and socioeconomic factors could lead to ethnic groups receiving treatment at a less favorable stage of the disease in hospitals with worse structures, resulting in an overall disparate outcome. Such hospital-level factors include, among others: geographical locations (i.e., hospitals serving predominantly minorities), time to admission to the ICU, adherence to and quality of established sepsis protocols or 'bundles' and time to first antibiotic therapy [29]. In a recent study, Rusch et al. analyzed over 4 million patients and found that treatment in predominantly minority-serving hospitals resulted in significantly higher in-hospital mortality for all races than in non-minority-serving hospitals [26]. However, they also found that being African American, in contrast to being Hispanic or of another ethnic background, was not associated with a higher risk of in-hospital mortality [26].

Conversely, Vazquez Guillamet et al. did not observe relevant differences in the management and outcomes of critically ill patients with sepsis of different ethnic backgrounds [17]. These discrepancies suggest that different ethnic groups receive different hospital treatment, potentially leading to varied outcomes. Additionally, ethnic-specific triage decisions could contribute to outcome differences [30]. For example, it has been shown that African American patients receive more aggressive end-of-life care and are more likely to be admitted to the ICU at older ages [30]. However, our study is inadequate to investigate such possible factors leading to disparity in care before admission to the ICU. Our data is limited to patients being already treated in the ICU. We lack information on triage decisions and individual patients' socioeconomic status before admission. We acknowledge this as a limitation of our study and reference another publication that specifically evaluates this question [17].

Secondly, cultural factors, such as racism, socioeconomic factors, or different insurance, could cause individuals of different ethnicities to receive different treatment within a hospital system, potentially leading to varied outcomes. However, neither other authors nor our study found evidence of such unethical behavior [17]

Thirdly, members of different ethnic groups might per se have different risks for a poor outcome in case of sepsis. This could be due to genetic factors or, more importantly, to different baseline risk distributions due to pre-existing conditions (obesity, cardiovascular disease, etc.). These factors may explain the mortality disparity observed by Chaudhary et al. [31]. Considering these possibilities, we examined the baseline characteristics of our study's patients and found differences in age and initial SOFA scores. However, although statistically significant, the differences in SOFA scores were of questionable clinical relevance, with a median of only one point. The higher SOFA score in African American patients aligns with a very recent study by Miller and colleagues [32]. They used the same eICU dataset as we did and found that the SOFA score overestimates the severity of the disease in African American patients. This could be due to the inclusion of serum creatinine in the calculation for the SOFA score.

Several other limitations of our study need to be considered: Given the observational nature of our data, inherent limitations include the lack of randomization, which does, as stated before, not allow for any causal conclusions, but rather careful consideration and interpretation of associations. Our data may include a certain selection bias due to only including patients already admitted to the ICU, lack of information on patients presenting with sepsis in the emergency department but not being admitted to the ICU, lack of information on the ethnicity-specific incidence of sepsis and septic shock in the overall population, and lack of data on any intensive care triage processes and therapy limitations. Additionally, information on the functional status of the patients (frailty) is missing in our analysis. We did, however, correct for the quantitative extent of comorbidities and found no evidence for a distinct outcome between races. Unfortunately, the number of patients in the minority groups is small (especially in the Asian American and Native American groups) compared to our the reference group. It is essential to know that in eICU the information about "race" is self-reported, and eICU does not consider patients from mixed ethnic backgrounds. To conduct our study, we utilized eICU, a database that only contains data from 2014 and 2015 [19]. As a result, we defined sepsis using the APACHE IV criteria [18]. While the Sepsis 3 definition for sepsis and septic shock has since been established [32], it cannot be easily applied to eICU studies. Overall, we believe that this analysis of a large real-world database encompassing multiple US hospitals does quite reliably rule out any influence of ethnicity as a factor for survival in sepsis, given (as in this analysis) that patients are treated uniformly in the ICU. Our study underscores the importance of considering the impact of socioeconomic differences rather than race when assessing health disparities. Further research should evaluate primarily health economic and public health interventions in this regard, whereas the ancient concept of "races" should be abandoned [33, 34].

## Conclusion

After admission to the ICU, there are no ethnic differences in treatment and outcomes for septic patients. Therefore, distinct outcomes among critically ill patients of different ethnicities are a public health, rather than a critical care challenge.

## Data Availability

All data relevant for this study will be given by the authors upon specific request. Patients or the public WERE NOT involved in the design, or conduct, or reporting, or dissemination plans of our research.

## Abbreviations

95%CI: 95% Confidence Intervals
aOR: Adjusted Odds Ratios
APACHE score: Acute Physiology And Chronic Health Evaluation
BMI: Body Mass Index
eICU: Electronic ICU Collaborative Research Database
GEE: Generalized Estimated Equation
GI: Gastro Intestinal
HIPAA: Health Insurance Portability and Accountability Act
ICU: Intensive Care Units
SD: Standard Deviation
SOFA score: Sequential Organ Failure Assessment.
US: United States of America
UTI: Urinary Tract Infection
